# Radiation-induced second malignancies after involved-node radiotherapy with deep-inspiration breath-hold technique for early stage Hodgkin Lymphoma: a dosimetric study

**DOI:** 10.1186/1748-717X-9-58

**Published:** 2014-02-18

**Authors:** Uwe Schneider, Marcin Sumila, Judith Robotka, Damien Weber, Günther Gruber

**Affiliations:** 1 Faculty of Science, Universtiy of Zürich, Zürich, Switzerland; 2 Institute for Radiotherapy, Witellikerstrasse 40, 8032 Zürich, Switzerland; 3 Radiation-Oncology, Animal Hospital, University of Zürich, Zürich, Switzerland; 4 Radiation Oncology, Geneva University Hospital, Geneva, Switzerland

## Abstract

**Background:**

To estimate the risk of radiation induced second cancers after radiotherapy using deep-inspiration breath-hold (DI) technique with three-dimensional conformal (3DCRT) and volumetric arc therapy (VMAT) for patients with Hodgkin’s lymphoma (HL).

**Methods:**

Early-stage HL with mediastinal and supraclavicular involvement was studied using an Alderson phantom. A whole body CT was performed and all tissues were delineated. The clinical target volumes and planning target volumes (PTV) were determined according to the German Hodgkin study group guidelines. Free-breathing (FB) technique and DI technique were simulated by different safety margins for the PTV definition. In both cases, 30 Gy in 15 fractions was prescribed. Second cancer risk was estimated for various tissues with a second cancer model including fractionation.

**Results:**

When compared with FB-3DCRT, estimated relative life time attributable risk (LAR) of cancer induction after DI-3DCRT was 0.86, 0.76, 0.94 and 0.92 for breast, lung, esophagus and stomach, respectively. With DI-VMAT, the corresponding values were 2.05, 1.29, 1.01, 0.93, respectively. For breast cancer, the LAR observed with DI-VMAT was not substantially distinguishable from the LAR computed for mantle RT with an administered dose of 40 Gy.

**Conclusions:**

This study suggests that DI may reduce the LAR of secondary cancers of all OARs and may be a valuable technique when using 3DCRT. Conversely, VMAT may increase substantially the LAR and should be cautiously implemented in clinical practice.

## Background

Early-stage Hodgkin’s lymphoma (HL) patients treated with a combination of chemotherapy and radiotherapy have an excellent clinical outcome, with overall survival of approximately 90% [1]. As a result, increased attention has focused on long-term toxicity of HL treatment. Treatment-induced complications, including but not limited to endocrinopathy, cardio-vascular disease and secondary malignancies, can induce substantial morbidity and significantly affect the quality of life of HL survivors [2]. With increasing follow-up after treatment, mortality from second cancers ultimately surpasses that of HL [3]. Consequentially, the current radiation therapy planning paradigm for the treatment of HL, is a reduction of field size, as randomized prospective trials have shown that regional therapy (i.e. involved-field radiotherapy, IFRT) is as effective as extended-field radiotherapy. To capitalize the potential decrease of toxicity with IFRT, further field reduction, including only the involved-nodes (INRT) in the target volume, has been advocated. INRT should further decrease the late radiation-induced toxicity [4,5] and diminish the likelihood of secondary tumors [6].

Another possibility to further reduce the target volume and thus the irradiated volume is the use of gating techniques such as deep-inspiration breath-hold (DI). DI techniques can be implemented with intensity modulation techniques, such as volumetric arc therapy (VMAT). Recently it was shown [7] that radiation exposure of the coronary arteries, heart, and lungs in patients with mediastinal Hodgkin’s lymphoma was greatly reduced using DI with intensity modulated radiotherapy (IMRT) and/or VMAT. The greatest benefit was obtained for tumors in the upper part of the mediastinum [7]. In another study Charpentier et al. [8] have shown that the combination of DI and parallel-opposed beam radiotherapy can significantly reduce lung and heart dose, however, has the potential to increase breast dose in females.

The aim of this phantom-based-study was to perform a second cancer risk comparison, using three-dimensional conformal radiotherapy (3DCRT) and VMAT and two planning paradigms for HL, namely free-breathing (FB) INRT and DI INRT.

## Methods

### Patient model

The Alderson Rando Phantom with a 200 ml breast attachment was used to represent a young female Hodgkin patient. It was decided to use a phantom instead of real patient data since a high resolution whole body CT could be performed. In addition the Alderson phantom is used for detailed stray dose measurements in our institution [9,10] which are employed later in this work to reconstruct a precise dose distribution. It was our study funding hypothesis that the advantage of a precise high resolution dose distribution as the basis for second cancer induction calculations outperforms the disadvantage of real patient data.

The 3D cube was reconstructed in a 512 × 512 matrix with a resolution of 0.12 cm in the x,y plane and a z-axis resolution of 0.5 cm. The phantom was manually segmented as a computerized 3-dimensional volume array, modeling of all major internal structures of the body.

### Target volume delineation

The target volumes, were delineated on the bases of real patient data according to the German Hodgkin Study Group (http://www.ghsg.org) guidelines [11]. More specifically, the clinical tumor volume (CTV) was the initial morphological volume of the initial mediastinal and/or supraclavicular mass with a significant PET uptake observed in the pre-chemotherapy diagnostic CT and PET. The real patient data were matched to the CT of the Alderson Rando phantom for delineation of the target structures. Delineation was performed by the same radiation oncologist (MS) who did the segmentation of the healthy structures. Four different lymph node involvements were studied: mediastinal both sides, mediastinal and supraclavicular both sides, mediastinal both sides and supraclavicular left, and mediastinal left.

PTVs for the four different involvements were created on the bases of the German Hodgkin disease study protocols by using 3 cm margins in the cranial-caudal direction and 1.5 cm in the lateral direction for FB [11]. DI was simulated by reducing the margins according to Wong et al. [12] to 1 cm and 0.5 cm, respectively. The corresponding volumes for FB and DI were 611.9 cm^3^ and 322.2 cm^3^ for mediastinal both sides, 1153.6 cm^3^ and 433.6 cm^3^ for mediastinal and supraclavicular both sides, 882.7 cm^3^ and 375.6 cm^3^ for mediastinal both sides and supraclavicular left, and 291.9 cm^3^ and 126.1 cm^3^ for mediastinal left.

In addition to the INRT targets, IFRT targets and classical mantle fields were defined to test the second cancer calculations with historical data. The planning target volume for IFRT was derived from the Ann Arbor Staging system.

### Treatment planning

**Figure 1:**
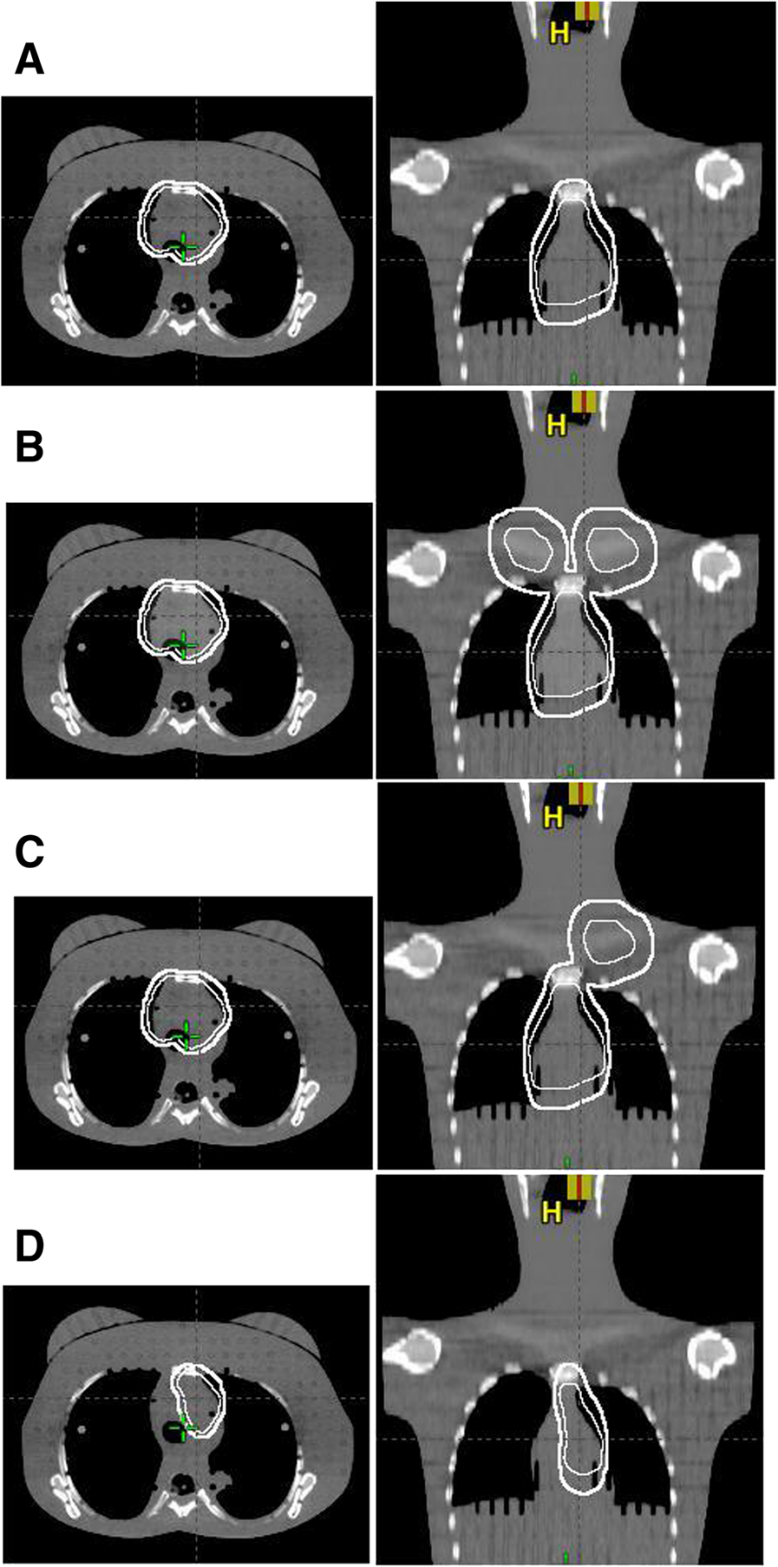
**Illustration of the four INRT-PTV in a transversal (left) and frontal (right) slice: (A)** mediastinal both sides, **(B)** mediastinal and supraclavicular both sides, **(C)** mediastinal both sides and **(D)** supraclavicular left, and mediastinal. The white bold line shows the FB target and the white line the DI target.

Both FB-INRT and DI-INRT were planned to deliver 30 Gy in 2 Gy fractions to PET- & CT-defined target volumes (Figure 1). Treatment planning was performed on the basis of the German Hodgkin disease study protocols (http://www.ghsg.org). We used for treatment planning the Eclipse External Beam Planning system version 10.0 (Varian Oncology Systems, Palo Alto, CA) using the AAA-algorithm (version 10.0.28) with corrected dose distributions for head-, phantom- and collimator-scatter [9,10]. All 3DCRT plans were calculated with 6 MV photons and consisted of two isocentrical anterior-posterior (AP/PA) opposed fields. In addition volumetric arc intensity modulated treatment (VMAT) was planned for the DI target volumes using two 360° rotation for the bi-lateral involvements and two 180° arcs for the homo-lateral involvements with the dose constraints listed in [4]. The corresponding conformity index according to RTOG for FB-VMAT and DI-VMAT was 1.00 and 1.03 for mediastinal both sides, 1.00 and 1.05 for mediastinal and supraclavicular both sides, 0.97 and 0.99 cm^3^ for mediastinal both sides and supraclavicular left, and 0.99 and 1.04 for mediastinal left.

For the comparison with historical data a classical mantle field irradiation was planned on the basis of the review by Hoppe [13]. The fields were arranged with 6 MV radiation and equal field weights from 0° and 180° at a distance of 100 cm (SSD). The dose prescription of the mantle field was 40 Gy in 2 Gy fractions. In addition, also with 40 Gy prescription, several IFRTs (Supraclavicular/neck, Axillary + Mediastinal/homolateral, Axillary + Mediastinal/bilateral, Axillary no Mediastinal involvement) were planned.

Differential dose-volume histograms (DVH) were extracted from the treatment planning system and were used for the second cancer risk calculations.

### Estimation of second cancer risk

The carcinogenesis model [14-16] used in the estimation of the risk of second primary malignancies emphasizes the cell kinetics of radiation induced cancer by mutational processes. Briefly, the model integrates cell sterilization processes described by the linear-quadratic model and repopulation effects. The model parameters were obtained by fits to several epidemiological, cancer specific carcinogenesis data [17-19] for carcinoma and sarcoma induction. Radiation induced lung cancer estimates were determined with the obtained model parameters from [17], breast cancer estimates from [18] and esophagus, stomach, thyroid, soft tissues and bone from [19]. Soft tissue sarcoma induction was estimated on the basis of the DVH for all normal tissues without the segmented organs and bone. For bone sarcoma induction, the DVH of the complete bone structure was used.

From the DVHs of the structures of interest, organ equivalent dose was calculated [15]. Organ equivalent dose was converted to excess absolute risk for a western population for each organ and for all organs together [14]. The resulting life time cancer risk for a patient from the specific radiotherapy treatment was determined by life time attributable risk (LAR) according to Kellerer et al. [20] by an integration of excess absolute risk from the age at exposure to the life time expectancy. LAR was calculated either as a function of age at exposure or was computed for one specific age at diagnosis of HL (20 years).

LAR is a life time risk and not applicable to epidemiological studies which include subjects with limited follow-up time. Therefore, cumulative risk is determined for these patients by taking into account the follow-up time instead of the life expectancy.

Base line risks for the different cancer sites were taken from Bray et al. [21] for the European union cohort.

### Epidemiological data

The second cancer model was tested with breast cancer data from two epidemiological studies from historical Hodgkin’s disease treatments. Hodgson et al. [22] studied the cumulative incidence for second cancers for specific attained ages and ages at Hodgkin’s disease diagnosis. They identified 850 excess second cancers from 18862 5-year Hodgkin’s survivors. They obtained cumulative risk for second breast cancer as a function of attained age for female Hodgkin patients diagnosed at the age of 20 [23]. We adjusted the data from Table 1 of Hodson et al. [22] to the baseline risks of the European Union and compared them with our findings.

**Table 1:** Lifetime attributable risk for DI-3DCRT and DI-VMAT relative to a free-breathing 3DCRT for INRT treatments with 30 Gy prescribed dose

**Cancer site**	**Mediastinal-both**	**Mediastinal-both & Supra-both**	**Mediastinal-both & Supra-left**	**Mediastinal-left**
**Breast**				
3DCRT	0.86	0.84	0.85	0.91
VMAT	2.05	1.82	1.89	1.93
**Lung**				
3DCRT	0.76	0.80	0.78	0.74
VMAT	1.29	1.11	1.17	1.55
**Esophagus**				
3DCRT	0.94	0.98	1.06	0.90
VMAT	1.01	1.12	1.15	1.00
**Stomach**				
3DCRT	0.92	0.91	0.92	0.93
VMAT	0.93	0.93	0.93	0.92
**Thyroid**				
3DCRT	0.89	0.61	0.62	0.93
VMAT	0.88	0.67	0.79	0.93
**Bone sarcoma**				
3DCRT	0.88	0.87	0.82	0.88
VMAT	0.62	0.58	0.60	0.63
**Soft tissue sarcoma**				
3DCRT	0.89	0.83	0.83	0.90
VMAT	0.75	0.65	0.69	0.78
**All cancers**				
3DCRT	0.82	0.82	0.82	0.86
VMAT	1.59	1.36	1.43	1.63

The second comparison was performed with the results of the study of De Bruin et al. [24]. They accessed the long-term risk of breast cancer after treatment for Hodgkin’s lymphoma and focused on the volume of breast tissue exposed to radiation. They found that mantle field irradiation (involving the axillary, mediastinal, and neck nodes) was associated with a 2.7-fold increased risk (95% CI, 1.1 to 6.9) compared with similarly dosed (36 to 44 Gy) mediastinal irradiation alone (see Table 2).

**Table 2:** Test of the applied second cancer model by comparison of modeled and observed relative breast cancer risk

**Planning paradigm**	**Used treament plans**	**Weighting according to # treated patients**	**Calculated relative risk (this work)**	**Observed relative risk from De Bruin et al. [**[24]**]**
Mediastinal	Mediastinal	109	1	1
Mantle	Mantle field alone	637	2.2	2.7 (1.1-6.9)
Other Supradiaphragmatic	Supraclavicular/neck	34		
	Axillary + Mediastinal/homolat	41		
	Axillary + Mediastinal/bilat	7		
	Axillary, no Media.	14		
	Total	96	1.0	0.9 (0.2-4.8)

Modeling was performed with a total dose of 40 Gy delivered in 2 Gy fractions.

## Results

### Modeled risk compared to epidemiological data

In Table 2 the observed excess risk from De Bruin et al. [24] of radiation induced breast cancer is listed relative to mediastinal IFRT. In the same table the modeled relative LAR is listed for a age at exposure of 20 years.

**Figure 2:**
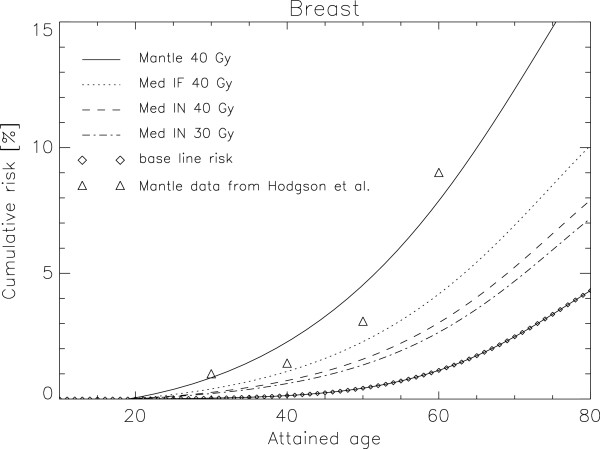
**Plot of cumulative absolute risk of radiation induced breast cancer as a function of attained age for a 20 year old female patient.** The triangles represent the data from Hodson et al. [23] for mantle field irradiation in comparison with the modelled data for 40 Gy as the solid line. The dotted line marks mediastinal IFRT with the prescribed dose of 40 Gy. A volume reduction with 40 Gy INRT is shown as the dashed line and an additional dose reduction to 30 Gy is represented by the dashed-dotted line. Baseline risk is plotted as the diamonds.

Cumulative absolute risk of radiation induced breast cancer as a function of attained age is plotted in Figure 2 for a female patient with an age of exposure of 20 years. The triangles represent the data from Hodson et al. [22] for mantle field irradiation in comparison to the modelled data for 40 Gy as the solid line. The dotted line marks mediastinal IFRT with the prescribed dose of 40 Gy. If INRT is applied to reduce further the irradiated volume but keeping the dose at 40 Gy the corresponding risk is shown as the dashed line in Figure 2. An additional dose reduction with INRT to 30 Gy is represented by the dashed-dotted line. The baseline risk is plotted as the diamonds.

The risk of second breast cancer induction was halved (age 20: 0.5, age 50: 0.5, age 80: 0.6) with the change from mantle field irradiation to the treatment of HL by reducing the field size and keeping the dose constant (Figure 2). Further reduction of field size by applying INRT has the potential to reduce breast cancer induction by approximately 30%. An INRT dose reduction from 40 Gy to 30 Gy could reduce radiation risk further by 10%.

### Potential of DI radiotherapy

DI 3DCRT has the potential to further reduce breast cancer risk by 15% when mediastinal lymph nodes are treated. However, VMAT increased the cumulative absolute risk of breast cancer induction by 100% with DI mediastinal INRT.

Table 1 details the LAR relative to FB- and DI-3DCRT/VMAT for breast, lung, esophagus, stomach, thyroid, soft tissue sarcoma and bone sarcoma for 30 Gy INRT. The excess relative risk is calculated for mediastinal treatment on both sides, mediastinal and supraclavicular RT on both sites, mediastinal on both sides combined with supraclavicular involvement on the left side and a homolateral mediastinal involvement of the left side. The excess relative risk is more or less independent of lymph node involvement. Radiotherapy using DI-3DCRT techniques have the potential to improve second cancer induction rates by more than 15% with a variation from 40% for thyroid to 0% for esophagus. Although, DI-VMAT can reduce soft tissue and bone sarcoma risk by 30-40%, overall a 50% increase of LAR of solid cancer induction can be expected. This is due to the low absolute risks for sarcoma induction.

**Figure 3:**
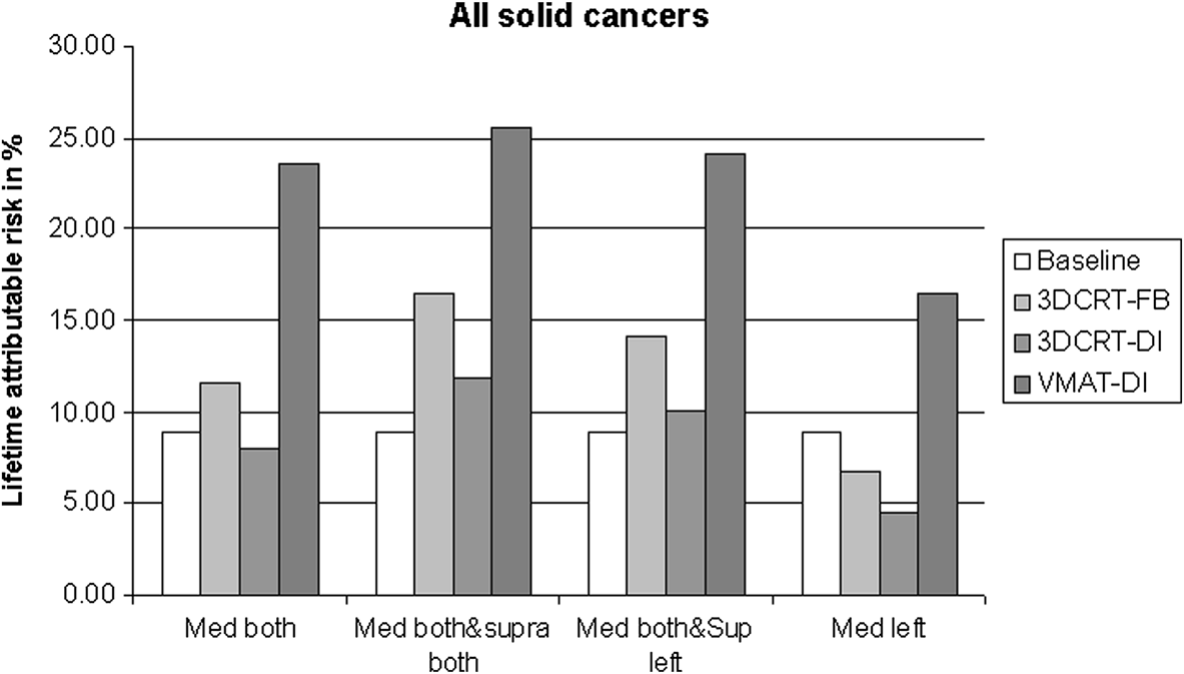
**Plot of LAR in% for all solid cancers analysed in this work.** FB-3DCRT is compared to DI-3DCRT and DI-VMAT for the lymph node involvements mediastinal both sides, mediastinal and supraclavicular both sites, mediastinal both sides combined with supraclavicular involvement on the left side and a homolateral mediastinal involvement of the left side.

The absolute life time second cancer risk (LAR) in% for a patient treated at the age of 20 is plotted in Figure 3 for the different lymph node involvements. Clearly DI-3DCRT has the potential to decrease the risk of cancer induction. However DI-VMAT is, regarding to secondary malignancy, the inferior treatment option.

**Figure 4:**
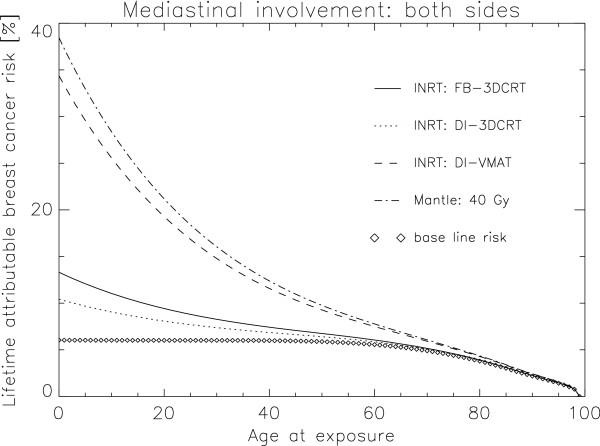
**LAR for breast cancer plotted as a function of age at exposure for a mantle field treatment with 40 Gy (dashed-dotted line), a FB-3DCRT treatment at 30 Gy (solid line), a DI-3DCRT at 30 Gy (dotted line) and a DI-VMAT treatment at 30 Gy (dashed line).** Baseline risk is plotted as the diamonds.

In Figure 4 LAR for breast cancer is plotted as a function of age at exposure for a mantle field treatment with 40 Gy (dashed-dotted line), a free-breathing 3DCRT treatment at 30 Gy (solid line), a DI-3DCRT at 30 Gy (dotted line) and a DI-VMAT treatment at 30 Gy (dashed-dotted line). Two effects can be observed. First, in absolute terms, DI-VMAT has a detrimental effect on cancer induction, even with a INRT paradigm, as the second cancer risk is almost as large as with a large mantle field irradiation. Secondly, age of exposure is an extremely important parameter regarding radiation induced cancer. One result of the calculations (Figure 4) is that LAR of breast cancer is more than two times larger for a HL patient treated at the age of 20 than for a 50 year old patient.

## Discussion

Late toxicities, especially radiation induced second cancers, are of major concern in patients treated for Hodgkin’s lymphoma [22,24]. To reduce the incidence of second cancer induction after radiation therapy, two possibilities can be explored: reducing the total radiation dose and reducing the volume (i.e. IFRT, INRT, DI). Our data suggest that volume reduction is the most effective way to reduce the number of secondary malignancies. LAR for a 20 year old patient relative to a historical mantle treatment is: 0.61 for IFRT 40 Gy, 0.55 for IFRT 30 Gy and 0.45 for INRT 30 Gy (Figure 2). A further reduction of the treated volume can be achieved by using deep-inspiration breath-hold technique which can reduce further cancer risk to 0.38 relative to a mantle field treatment.

When DI techniques are used to treat Hodgkin’s patients it is also possible to use intensity modulation techniques. Goodman et al. [25] published their experience with IMRT in HL and non-HL involving the mediastinum. They demonstrated that IMRT was able to reduce the dose delivered to the heart and the lungs in comparison with conventional parallel-opposed fields and 3DCRT. Recently Paumier et al. [7] found similar results: radiation exposure of the coronary arteries, heart, and lungs in patients with mediastinal Hodgkin’s lymphoma was greatly reduced using DI with IMRT. Despite the obvious advantage to reduce side effects related to the heart and the coronary arteries in Hodgkin’s patients, an open question is the impact of intensity modulation techniques on the incidence of second malignancies. We could show that DI-VMAT has the potential to increase life time attributable risk relative to a historical mantle treatment to 0.91 and thus nearly outbalance the advantages which was gained with the current paradigm of volume and dose reduction. Regarding second cancer induction VMAT techniques have solely an advantage regarding sarcoma induction, as sarcomas occur usually in heavily irradiated volumes and the volumetric modulation technique is modifying the dose distribution from high-dose-small-volume to low-dose-large-volume. However, since the incidence of sarcomas is small when compared to carcinoma induction, the impact on the total risk for radiation induced cancer is small.

Although it was shown in this work that DI-VMAT is inferior to classical parallel opposed treatment techniques with regard to second cancer induction it should be noted that VMAT techniques, gated or non-gated, ameliorate the deterministic toxicity profile. It was shown [26-28], that the application of VMAT techniques can result in better dose spearing of the thyroid gland, the heart and the coronary ostia.

The results of this study are based on a model that, while improved over older secondary malignancy models by including fractionation, excludes potential confounding factors that may affect secondary lung and breast malignancy risk. These potential factors include family history, chemotherapy use and environmental factors like smoking.

In this work it was decided to use an Alderson Rando phantom for treatment planning because it has the advantage that precise dose information including stray doses on a high resolution whole body CT can be used for second cancer calculations. However, the use of a phantom instead of patient data has two disadvantages. First, a phantom is not accounting for the variation in anatomy between patients. Second, the simulated DI technique affects only the size of the PTV. However, the use of the DI technique allows a significant reduction in mediastinal volumes [7] due to the anatomical changes of free breathing when compared to deep inspiration. Thus, the additional sparing of organs at risk achieved with DI is not simply due to smaller PTVs. This additional volume reduction was not considered in this work and thus further patient studies are needed to explore second cancer induction in Hodgkin’s patients in more detail.

## Conclusion

The additional volume reduction by applying DI 3DCRT of HL patients has the potential to further minimize life time second cancer risk (LAR) to 0.38 relative to a historical mantle treatment. This is an additional 15% reduction due to the use of DI.

The combined use of VMAT with DI allows significant sparing of the organs at risk like the coronary arteries and the heart in comparison with 3DCRT. However, second cancer risk is substantially increased to 0.91 when compared to 40 Gy administered with a 2D mantle field technique. Thus the risk-gain due to volume and dose reduction is almost negatively balanced by the application of volumetric modulation techniques.

## Competing interests

The authors declare that they have no competing interests.

## Authors’ contributions

US designed the study, performed the risk estimates and wrote the manuscript. MS, DW and GG prepared the patient segmentation and treatment planning and helped writing the manuscript. JR performed treatment planning. All authors reviewed and approved the final manuscript.
